# Microscopic and molecular detection of piroplasms among sheep in Upper Egypt

**DOI:** 10.3389/fvets.2024.1373842

**Published:** 2024-05-27

**Authors:** Ahmed Kamal Dyab, Sara Abdel-Aal Mohamed, Fatma Mohamed Abdel-Aziz, Ahmed Gareh, Fathy Osman, Fatma A. Elgohary, Ehssan Ahmed Hassan, Noorah Alsowayeh, Hind Alzaylaee, Abd Al-Rahman S. Ahmed, Daniel Bravo-Barriga, Ehab Kotb Elmahallawy

**Affiliations:** ^1^Department of Parasitology, Faculty of Medicine, Assiut University, Assiut, Egypt; ^2^Department of Parasitology, School of Veterinary Medicine, Badr University in Assiut, New Nasser City, Assiut, Egypt; ^3^Department of Parasitology, Faculty of Veterinary Medicine, Assiut University, Assiut, Egypt; ^4^Department of Parasitology, Faculty of Veterinary Medicine, Aswan University, Aswan, Egypt; ^5^New Valley Lab, Department of Parasitology, Animal Health Research Institute, Agriculture Research Center (ARC), New Valley province, Egypt; ^6^Department of Hygiene and Zoonoses, Faculty of Veterinary Medicine, Mansoura University, Mansoura, Egypt; ^7^Department of Biology, College of Science and Humanities in Alkharj, Prince Sattam Bin Abdulaziz University, Alkharj, Saudi Arabia; ^8^Department of Zoology, Faculty of Science, Suez Canal University, El-Sheikh Zayed, Ismailia, Egypt; ^9^Department of Biology, College of Science in Al-Zulfi, Majmaah University, Al-Majmaah, Saudi Arabia; ^10^Department of Biology, College of Science, Princess Nourah Bint Abdulrahman University, Riyadh, Saudi Arabia; ^11^Department of Natural Resources, Faculty of African Postgraduate Studies, Cairo University, Giza, Egypt; ^12^Departamento de Sanidad Animal, Grupo de Investigación en Sanidad Animal y Zoonosis (GISAZ), Universidad de Córdoba, Córdoba, Spain; ^13^Department of Zoonoses, Faculty of Veterinary Medicine, Sohag University, Sohag, Egypt

**Keywords:** sheep, piroplasm, *Babesia*, *Theileria*, microscopic, molecular, prevalence, Egypt

## Abstract

**Introduction:**

Blood parasites pose a significant threat to livestock production in southern Egypt, yet there is a scarcity of information regarding their circulation and epidemiology in sheep in this region. This study aimed to investigate the seroprevalence of blood parasite infections in sheep in Assiut governorate, Upper Egypt.

**Methods:**

A total of 400 blood samples were collected from sheep of varying ages and genders. The preliminary screening for the presence of piroplasms, mainly *Babesia* and *Theileria* spp., via microscopic examination, followed by investigation of the potential risk factors linked with the exposure to infection. Moreover, molecular identification of both parasites on some of positive samples was performed using PCR targeting *Babesia* 18S rRNA and *Theileria annulata* Tams1 gene.

**Results:**

The microscopic examination revealed that among the examined sheep, there was an overall prevalence of blood parasites at 44% (176 out of 400), with *Babesia* spp. observed in 14% (56 out of 400) and *Theileria* spp. in 30% (120 out of 400). Furthermore, the infection rate was non-significantly higher in young animals (50%) compared to adults (38.5%) (*P* = 0.246). Male sheep exhibited a significantly higher vulnerability to both parasites' infection (63.3%) compared to females (35.7%) (*P* = 0.011). Interestingly, the prevalence of both blood parasites was significantly higher during the cold season (66.1%) compared to the hot season (15.9%) (*P* = < 0.001). The molecular analysis identified the presence of *Babesia ovis* and *Theileria annulata* among a subsample of the positive sheep's bloods films. The identified species were recorded in the GenBank™ databases and assigned specific accession numbers (OQ360720 and OQ360719 for *B. ovis*), and (OP991838 for *T. annulata*).

**Conclusions:**

Taken together, this study confirms a high prevalence of piroplasmosis and offers epidemiological and molecular insights into blood parasites in sheep from Upper Egypt, highlighting the importance of detecting these parasites in various hosts and their competent vectors (ticks).

## 1 Introduction

Sheep industry is a significant economic asset due to the animals' resilience to food shortages and weather fluctuations (1). In Egypt, sheep and goats play a vital role in providing meat to humans, along with serving as sources for wool production and utilizing their manure as a fertilizer for soil (2). Nevertheless, sheep are vulnerable to numerous haemoprotozoan diseases that can impact their health and productivity. Among various others, *Theileria* spp. and *Babesia* spp., responsible for theileriosis and babesiosis, respectively, stand out as highly economically significant tick-borne diseases transmitted to sheep (3). These haemoprotozoa are part of the broader classification belonging to the Class Sporozoa, Order Piroplasmida, and families Babesiidae and Theileriidae (4). In terms of transmission, *Theileria* and *Babesia* are biologically transmitted by hard ticks in tropical and subtropical regions globally (5). Both diseases frequently occur between May and September, coinciding with the periodic movement of ticks (6). Regarding the life cycle of *Babesia* (7), the organism undergoes asexual division within a mammalian red blood cell (RBC), after which the daughter merozoites rupture the host cell to infect new erythrocytes. Ticks become infected by consuming erythrocytes containing merozoites. Sexual reproduction (gametogony) takes place in the tick gut, followed by asexual sporogony in its salivary glands. Subsequently, the development of infective stages (sporozoites) occurs in the salivary glands of infected ticks. This cycle can persist throughout the animal's lifespan depending on the host's immunity. Regarding their transmission, two distinct routes of transmission are involved: transstadial transmission from one stage of the tick's life cycle to the next and transovarial transmission (8–10). The life cycle of *Theileria* closely resembles that of *Babesia*, involving sexual gametogony followed by asexual sporogony in the tick gut. Ultimately, the inoculated sporozoites invade lymphocytes in the infected host, prompting continuous division as lymphoblasts through a process known as cellular transformation. This results in parasite replication, and the infected cells become widely distributed throughout the host's lymphoid system. After 2–3 weeks, cytotoxic T-cells damage parasitized lymphoblasts. Simultaneously, the parasites initiate an attack on erythrocytes, reproducing asexually within them. Ticks become infected through the ingestion of these infected erythrocytes. Transstadial transmission is the only established mode of transmission in *Theileria* spp., with no evidence of discernible transovarial transmission (11). Among others, *Rhipicephalus* (*Boophilus*) *annulatus* and other members genus *Hyalomma*, such as *H. marginatum* and *H. excavatum*, are considered the most predominant ticks responsible for transmittion of piroplasmosis to sheep in Egypt (12, 13).

Ovine babesiosis is a significant tick-borne disease affecting small ruminants, caused by various species such as *B. ovis, B. motasi, B. crassa, B. taylori*, and *B. aktasi*, among others (6, 7, 10–12, 14–16). Taken into account, the most pathogenic species in sheep is *B. ovis* that causes malignant ovine babesiosis with fever, hemolytic anemia, hemoglobinuria, icterus and possible death others (6, 7, 10–12, 17, 18). Hemoglobinuria is uncommon, but it can occur in the advanced stages of the disease, leading to potential abortion in pregnant animals. Herds at risk can experience a significant mortality rate (19). Sheep with chronic infections typically display no symptoms, except for the presence of parasitemia and a lack of thriftiness. Recovered animals with latent infections usually possess immunity for a specific period, and there is no cross-immunity between the parasites (20). Theileriosis in small ruminants is caused by protozoan parasites of the *Theileria*, specifically including species such as *Theileria lestoquardi, T. ovis, T. recondita, T. annulata, T. separata, T. luwenshuni, T. uilenbergi, Theileria* sp. OT1 and *Theileria* sp. OT3 (7, 21). Common symptoms of theileriosis in sheep include cough, fever, lymphadenopathy, fatigue, and weight loss (22). Among others, *T. lestoquardi* is considered the most pathogenic species, leading to a malignant ovine theileriosis with a great morbidity and mortality proportion in sheep (23). Also, several previous investigations revealed that *Theileria* infection can result in abortion (24, 25). The diagnosis of ovine babesiosis and theileriosis can be achieved through clinical symptoms observation and microscopic examination of lymph or thin blood smears stained with Giemsa which remains the gold standard for diagnosis of the infection. However, it should be noted that relying solely on morphological characteristics for blood parasite detection (26, 27). On the other hand, molecular identification of these parasites offers greater sensitivity and specificity compared to alternative diagnostic methods (15). However, the cost of these later techniques poses a significant challenge, particularly in countries with limited resources.

It should be stressed that blood parasites pose a significant economic challenge in sheep production in Egypt, leading to losses in meat and milk production due to high parasitemia and mortality rates in infected animals (28, 29). Clearly, routine surveillance of these parasites among small ruminants in a particular area is essential for understanding the prevalence of these diseases and implementing effective control measures. While some prior studies have been conducted on ovine babesiosis and theileriosis in Egypt [Table 1; references (4, 30–32, 34–39)], there is a notable scarcity of research, especially the molecular work in the southern region of the country, namely Upper Egypt. Considering the importance of understanding factors affecting infection exposure within natural populations, it is crucial for comprehending host-parasite dynamics, predicting infection susceptibility, and maintaining biological equilibrium (40). While several factors elucidated differences in occurrence of the parasite and exposure to infection among host species, the relationship between hosts, ticks, and pathogens undergoes continuous changes, largely driven by ecological, climatic, and anthropogenic alterations. Various factors, including animal sex, age, herd management practices, seasonal variations, tick infestation, and herd size, have been associated with infection by blood parasite in animals. Additionally, spatio-temporal factors such as vector habitats, animal feeding systems, sanitation measures, and management practices significantly influence the epidemiology of these infections (41). As mentioned above, theileriosis and babesiosis are parasitic diseases of significant commercial importance, thereby playing a crucial role in the global trade of animals and animal products. The precise documentation of these parasites in Egypt is essential for understanding their epidemiology and classification, enabling effective control measures to mitigate the damage caused by their infections. Given the aforementioned details, the present study aims to investigate the prevalence and epidemiology of ovine babesiosis and theileriosis in Assiut Governorate, Upper Egypt, while also considering the potential associated risk factors. This assessment utilizes a combination of microscopic methods, followed by molecular identification of the parasites.

**Table 1:** Occurrence and genetic diversity of *Babesia* and *Theileria* spp. reported in sheep in Egypt.

**Area**	**Detection method**	**Frequency % (no. pos./total)**	**Species identified**	**Genotype (no.)**	**References**
Upper Egypt	CM	51.21 (ND) *Theileria ovis* 12.20 (ND) *Babesia sp*.	*Theileria ovis* *Babesia* sp.	ND	(30)
Upper Egypt	CM	20.00 (15/130) *Babesia* motasi 13.83 (13/130) *Babesia ovis* 14.6 (10/130) *Theileria ovis*	*Babesia motasi* *Babesia ovis* *Theileria ovis*	ND	(31)
Upper Egypt	CM	15.56 (54/347) *Theileria* spp.	*T. ovis, T. lestoquardi* and *T. annulata*	ND	(32)
Lower Egypt	CM	84.80 (ND)	*Babesia* spp. *Theileria* spp.	ND	(33)
Lower Egypt	PCR	10.19 (11/108) *B. bovis* 20.37 (22/108) *Theileria annulata* 6.48 (7/108) Mixed	*Babesia bovis* *Theileria annulata*	ND	(34)
Lower Egypt	CM	39 (117/300) (17) *Babesia* spp. (20) *Theileria* spp. (2%) Mixed	*Babesia* spp. *Theileria* spp.	ND	(4)
Lower Egypt	CM PCR	76 (76/100) 43(43/100)	*B. ovis, T. ovis*, and *A. ovis*	ND	(35)
Lower Egypt	CM IFAT	50.92 (55/108) *Babesia ovis* 71.3 (77/108) *Babesia ovis*	*Babesia ovis*	ND	(36)
Upper & lower Egypt	PM	0.95(1/105) *B. bovis* 1.90 (2/105) *B. bigemina*	*B. bovis B. bigemina*	ND	(37)
Upper & lower Egypt	CM PCR	21.70 (33/152) 36.80 (56/152)	*Theileria ovis* *Theileria lestoquardi*	ND	(38)
Upper & Lower Egypt	CM PCR	00 (0/115) 5.21 (6 /115)	*T. ovis* *T. lestoquardi*	ND	(39)

CM, Conventional Microscope; PCR, Polymerase Chain Reaction; IFAT, Indirect immunofluorescence antibody technique; ND, Not detected or non-determined; NS, Not specified.

## 2 Materials and methods

### 2.1 Study area and sample collection

**Figure 1:**
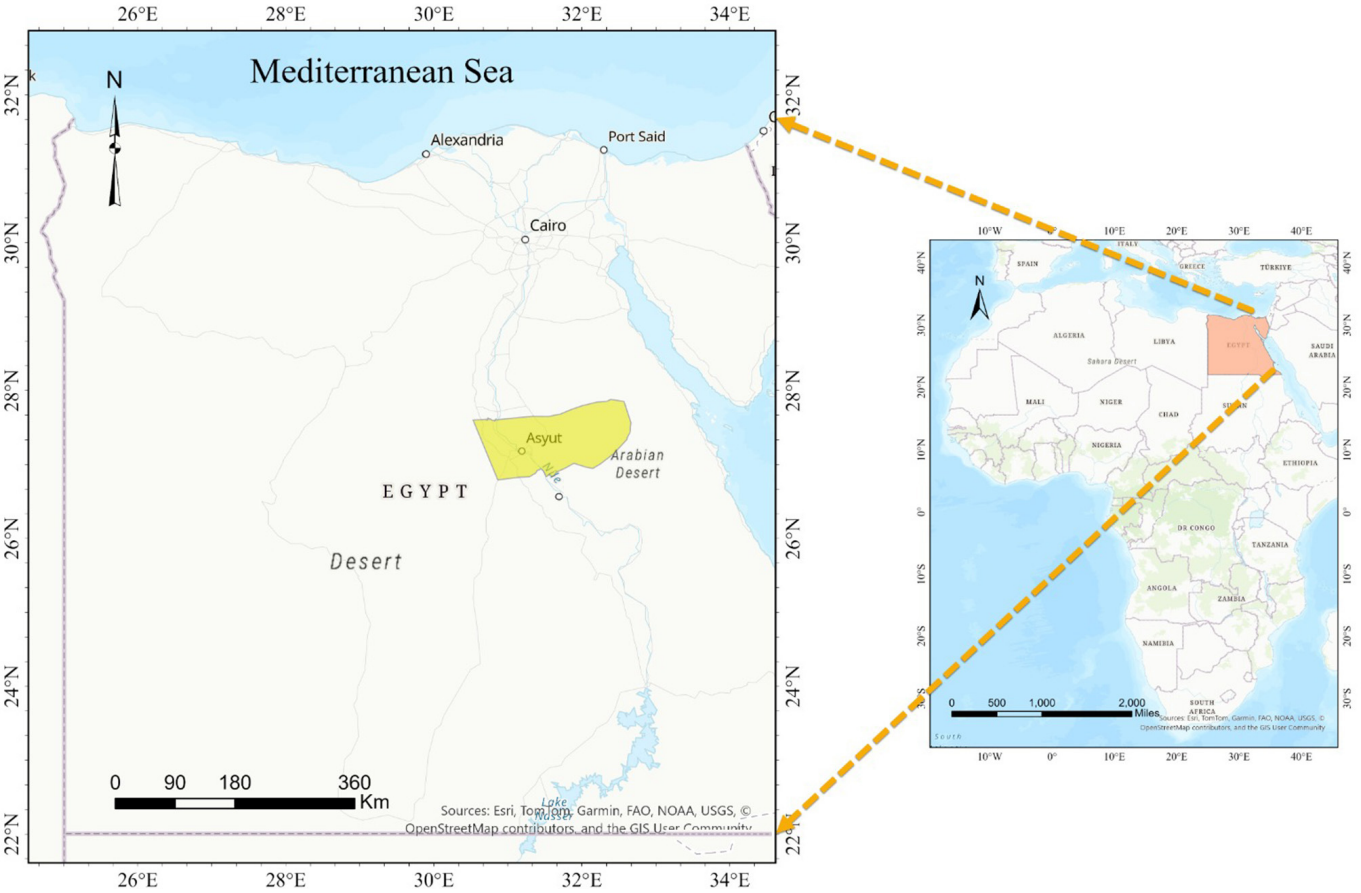
A map illustrating the geographic region being investigated.

The research comprised the random sampling of 400 blood samples of sheep from small-scale stakeholders participating in veterinary campaigns conducted between February 2022 and January 2023 across different locations in Assiut Governorate, Upper Egypt (Figure 1). The climate of the studied area exhibits notable temperature extremes, with June seeing the highest monthly average at 37.1z°C, while January experiences the lowest at 4.7°C. Additionally, humidity levels fluctuate considerably, ranging from a low of 25% in May to a peak of 52% in January. The sample size was determined based on a 95% confidence level to detect exposure to the parasites, assuming a maximum individual prevalence of 52% in Egypt (30, 42). The sampled sheep encompassed both sexes and various age groups. A 5 ml blood sample was drawn from the jugular vein of each animal into sterile vacutainer tube with ethylene-diamine tetra acetic acid (EDTA) (BD Vacutainer^®^, Franklin Lakes, NJ, USA, EUA). All the samples were refrigerated at 4–8°C. Once in the laboratory, we proceeded the preparation of blood films for examination under an ordinary microscope.

### 2.2 Microscopic examination

To prepare thin blood films, a drop of blood was placed on one end of a clean slide, and a spreader was slid at a 45° angle to touch the blood sample. The spreader was then gently and firmly moved, allowing the blood to follow behind and form a feathered edge. Subsequently, the thin film was air-dried, fixed in methanol for 5 min, and stained with a freshly prepared 10% Giemsa stain for 30 min. After staining, the film was washed with water and left to dry. Finally, the blood film was examined under a light microscope at × 100 (oil immersion) to detect blood parasites (43). The infected erythrocytes containing piroplasms or schizonts were identified as described in the literature (44, 45).

### 2.3 Molecular detection

#### 2.3.1 DNA extraction

The genomic DNA extraction was performed from some blood samples (*n* = 40), which tested positive for each parasite during microscopic examination, indicating a higher concentration of parasites per field, using a commercial DNA isolation kit (QIAamp DNA Mini Kit, 51304) following the manufacturer's instructions.

#### 2.3.2 PCR amplification and phylogenetic analysis

The amplification of *Babesia* through 18S rRNA gene and *Theileria* (*Tams1* gene) species was conducted following established protocols from previous studies (34, 46, 47). Preparation of PCR Master Mix for cPCR according to Emerald Amp GT PCR master mix (Takara), Code No. RR310Akit. The reaction mixture (25 μL) included 12.5 μL of Emerald Amp GT PCR mastermix (2x premix) (Code No. RR310A), 0.2 μM of each primer, 5 μL of DNA template and Millipore water was added to achieve a final volume of 25 μL. The set of primers and temperature and time conditions during PCR are shown in Supplementary Tables 1, 2 (48, 49), respectively. PCR amplicons were examined through 1.5% agarose gel electrophoresis, and for each 100 ml gel, 6 μL of GreenSafe stain (10 mg/mL) was applied. Additionally, a 100 bp ladder was utilized as a reference to identify target amplicon sizes. Gel documentation system captured images of the gel, and the data were subsequently analyzed using computer software. PCR products from some of the positive samples exhibited distinct and sharp bands were purified using the QIAquick PCR Product Extraction Kit (ID: 51304) from Qiagen, Valencia. For the sequencing reaction, the BigDye Terminator V3.1 Cycle Sequencing Kit from Perkinelmer was employed, followed by purification using a Centrisep spin column. PCR products with positive amplifications were purified and sequenced using the Applied Biosystems 3130 Genetic Analyzer. To achieve species-level identity, a score of >99% identity was considered using both BLASTn (https://blast.ncbi.nlm.nih.gov/Blast.cgi; Mega-BLASTn option) (34, 50). Multiple alignments were carried out with MAFFT version 7 (51). For maximum likelihood (ML) phylogenetic analyses, the choice of the best-fitting evolutionary model was based on those defined using JModeltest2 on the basis of the Akaike information criterion (51). Tree reconstruction was carried out with Mega 11 (52). The evolutionary history was deduced using the maximum likelihood method and the Tamura 3-parameter model (53). The percentage of trees in which the associated taxa clustered together is shown next to the branches. The tree is drawn to scale, with branch lengths measured in the number of substitutions per site. The phylogenetic trees were manipulated for display using FigTree v.1.4.2 (54).

### 2.4 Statistical analysis

The data collected in this study was analyzed using the Statistical Package for Social Sciences (SPSS) software program, version 26. The chi-square test was employed to examine and compare qualitative variables. Quantitative measures were expressed as means ± standard deviation (SD) and Median (Interquartile range). The prevalences were estimated as the ratio of positives from the total number of samples analyses, with the exact binomial confidence intervals of 95% (95% CI) based on the score method (55). Logistic regression analyses were conducted to evaluate both unadjusted and adjusted odds ratios (OR) along with their corresponding 95% confidence intervals (95% CI) to identify factors associated with infection. A significance level of *P* < 0.05 was considered significant, while *P* < 0.01 was deemed highly significant.

## 3 Results

### 3.1 Prevalence of blood parasites in surveyed sheep and associated risk factors

In the present work, out of the 400 blood samples examined by microscopic examination, 176 tested positive for blood parasites, resulting in an overall prevalence of 44%. The individual infection rates for *Babesia* spp. and *Theileria* spp. were 14% and 30%, respectively. Clinically the positive sheep were anemic, emaciated, having lacrimation, decreased appetite, and temperature may be normal or slightly increased. Furthermore, corneal opacity, enlargement of lymph nodes, and hemoglobinuria were recorded in some cases. In terms of age as potential risk factor, which is shown in Table 2, the overall prevalence of detected blood parasites was higher in young animals (50%) compared to adults (38.5%), although a significant association was not found (OR: 160; *P*-value: 0.246). As depicted in Table 2, the infection rate of *Babesia* spp. was 14% (16.7% in animals younger than 2 years and 11.5% in animals older than 2 years), while for *Theileria* spp., it was 30% (33.3% in animals younger than 2 years and 26.9% in animals older than 2 years). Concerning sex (Table 2), the overall prevalence of blood parasites in males was 63.3% (OR: 3.11, *P* = 0.01) which was higher than in females 35.7%. The significance of these findings is highlighted by the results observed in *Babesia*, revealing an odds ratio (OR) of 8.25 between males and females (Table 2). In contrast, Table 2 showed that *Theileria* had an OR of 0.46, indicating a different pattern of association between gender and infection rates in these two parasites. Table 2 also depicts a significant relationship between blood parasitic infection in relation to sex (*P* = 0.011). In terms of seasonality, a significant association was identified between blood parasitic infections and the seasons (*P* < 0.01) (OR: 10.29; *P* < 0.001) (Table 2). This pattern was notably consistent during the cold season for both *Babesia* spp. and *Theileria* spp., with odds ratios of 5.73 and 6.29, respectively (Table 2).

**Table 2:** Distribution of the overall prevalence of blood parasites, including *Babesia* and *Theileria*, infections according to the age group, sex, and sampling season of the surveyed sheep population (*n* = 400).

**Variable**	**Total examined**	**Infected (%)**	**95% CI**	**OR (95% CI)**	***P-*value**
**Total blood parasites**					
**Age**					
< 2 years	192	96 (50.00)	43.00–57.00	1.60 (1.08–2.38)	0.246
>2 years	208	80 (38.46)	32.12–45.23		
**Sex**					
Male	120	76 (63.33)	45.42–71.42	3.11 (1.99–4.85)	**0.011** ^*^
Female	280	100 (35.71)	30.33–41.49		
**Season**					
Cold	224	148 (66.07)	59.65–71.95	10.29 (6.31–16.79)	**< 0.001** ^**^
Hot	176	28 (15.90)	8.79–17.47		
***Babesia*** **spp**.					
**Age**					
< 2 years	192	32 (16.66)	12.06–22.58	1.53 (0.87–2.71)	0.460
>2 years	208	24 (11.54)	7.88–16.59		
**Sex**					
Male	120	40 (33.33)	25.43–42.17	8.25 (4.39–15.51)	**0.001** ^**^
Female	280	16 (5.71)	3.55–9.08		
**Season**					
Cold	224	48 (21.42)	16.56–27.26	5.73 (2.63–12.47)	**0.016** ^*^
Hot	176	8 (4.54)	2.32–8.71		
***Theileria*** **spp**.					
**Age**					
< 2 years	192	64 (33.33)	27.05–40.27	1.36 (0.88–2.08)	0.485
>2 years	208	56 (26.92)	21.35–33.33		
**Sex**					
Male	120	36 (30.00)	22.53–38.72	0.46 (0.29–0.71)	1.000
Female	280	84 (30.00)	24.93–35.61		
**Season**					
Cold	224	100 (44.64)	38.28–51.19	6.29 (3.86–10.74)	**< 0.001** ^**^
Hot	176	20 (11.36)	7.48–16.90		

Bolded values indicate statistical significance. ^*^p < 0.05; ^**^p < 0.001.

### 3.2 Morphological characteristics of blood parasites detected in sheep

**Figure 2:**
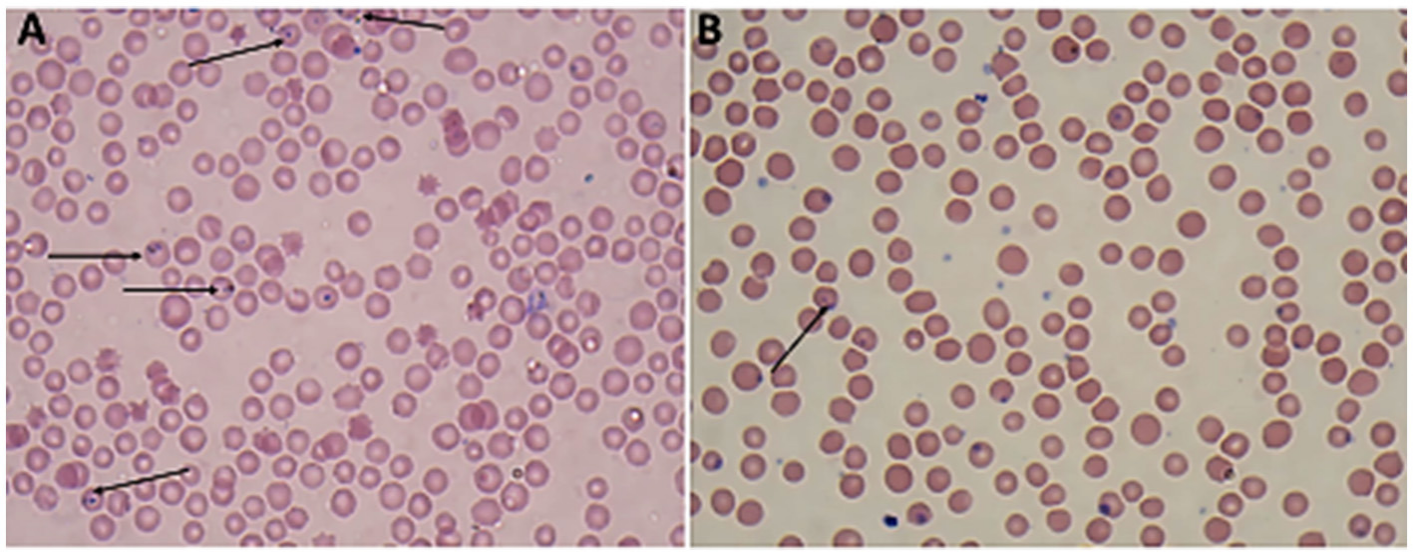
Thin blood film stained with Giemsa magnified × 100 (oil immersion lens) showing the blood protozoan parasites found in sheep **(A, B)**: paired Trophozoites of *Babesia* spp. (arrow).

**Figure 3:**
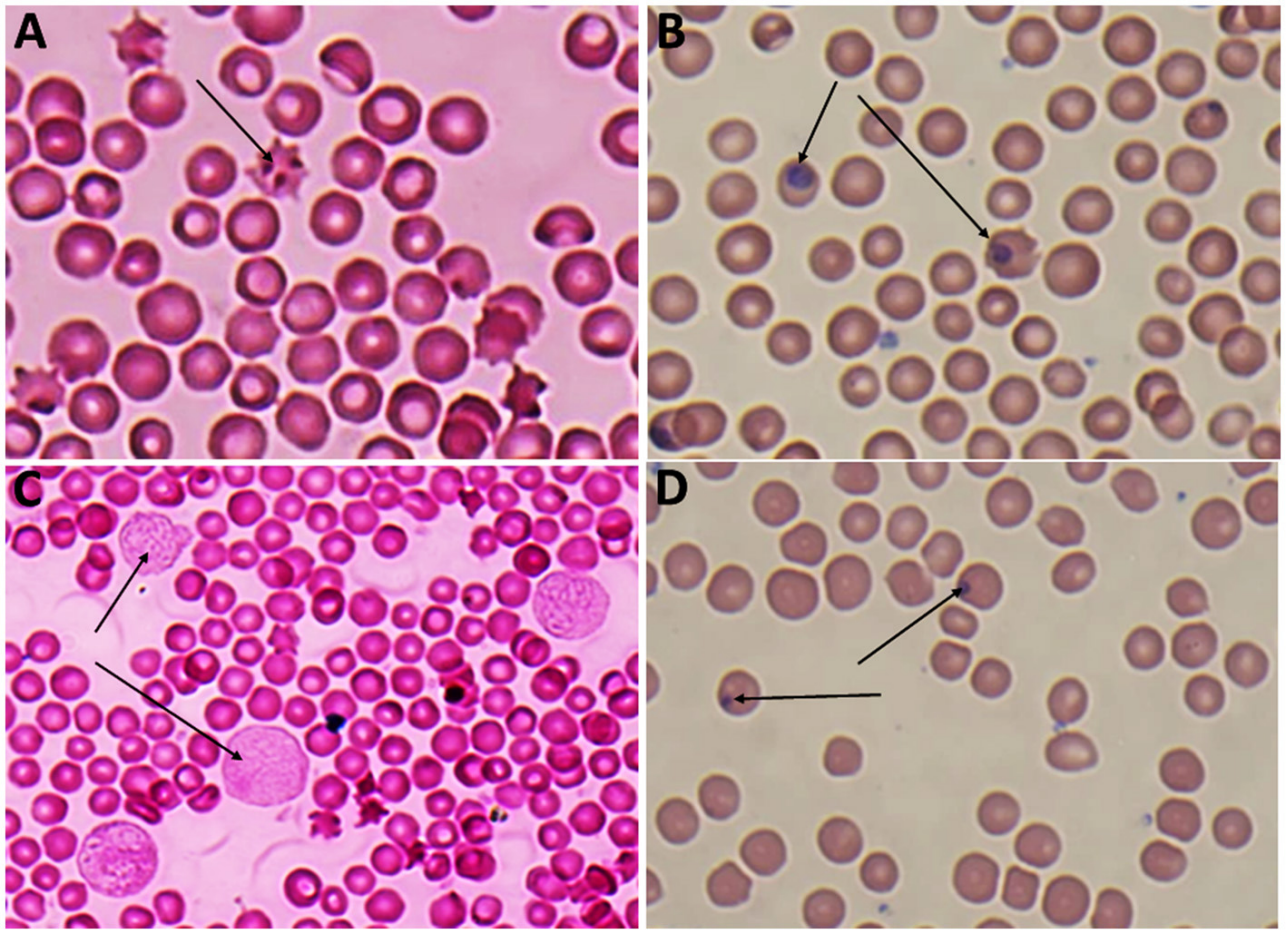
**(A)** Echincyte containing trophozoites of *Theileria* spp., x100 (oil immersion lens), **(B)** ring stage of *Theileria spp*., x100 (oil immersion lens), **(C)** multiple microshizonts of *Theileria* spp. x100 (oil immersion lens) (arrow), **(D)** small rod shape trophozoite of *Theileria* spp., x100 (oil immersion lens) (thin arrow), rounded shape trophozoite of *Theileria* spp., x 100 (oil immersion lens) (thick arrow).

Giemsa-stained blood smears of *Babesia* spp. showed erythrocytic stages in which intra-erythrocytic piroplasms are rounded or double pear-shaped typically located at the periphery of the infected host erythrocytes (Figure 2). Meanwhile, Giemsa-stained blood smears of *Theileria* spp. show erythrocytic stages, merozoites, schizonts and multiple microshizonts, which were intra-erythrocyte piroplasms of the small rod-shaped, ring, and rounded shaped forms present within lymphocytes (Figure 3). The infected red blood cells (RBCs) also displayed echinocyte-like protrusions on their surface, a common feature observed during *Theileria* infection.

### 3.3 PCR analysis

**Figure 4:**
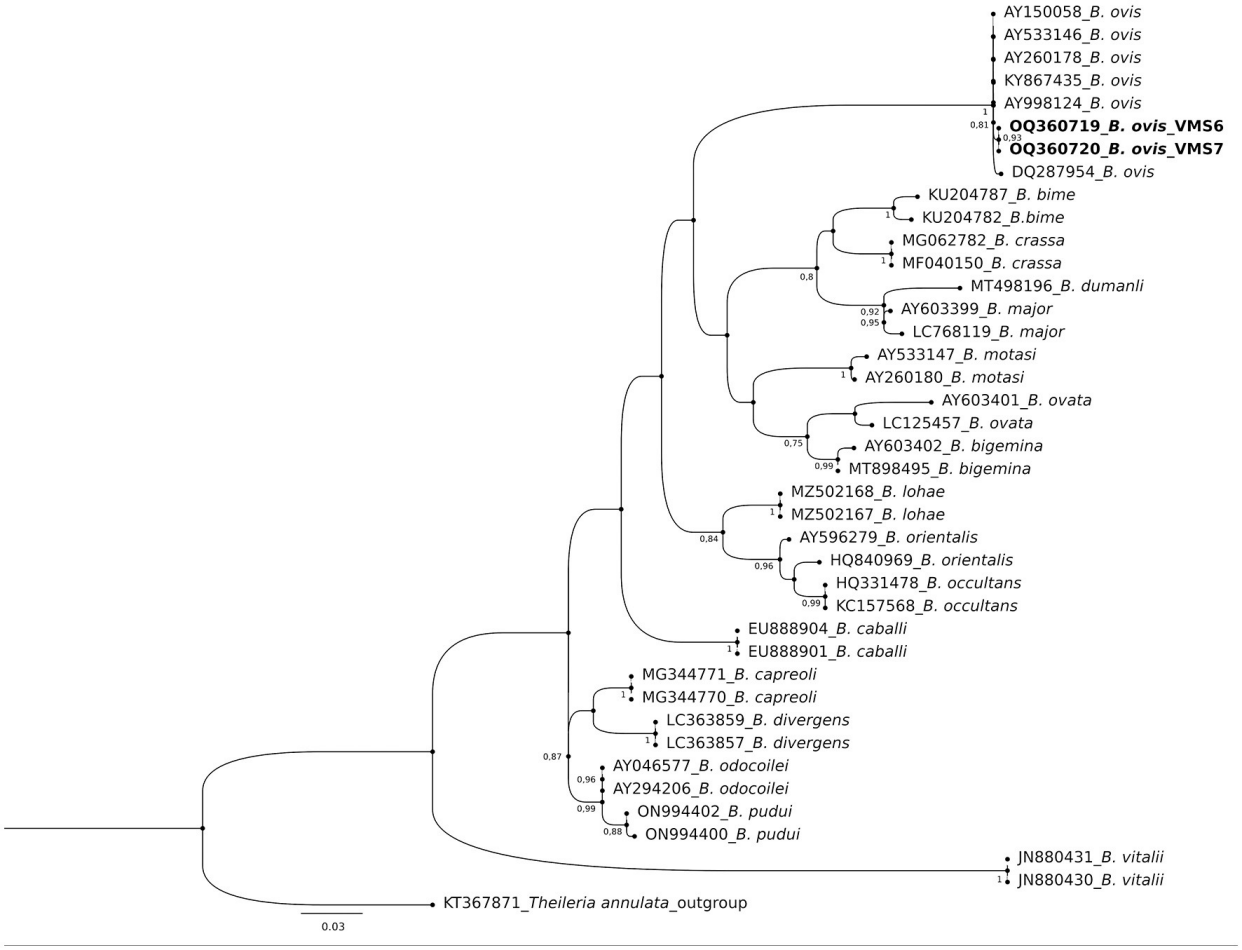
Phylogenetic relationships of *Babesia ovis* were inferred based on the analysis of 40 nucleotide 18S rRNA gene sequences using the Maximum Likelihood method and Kimura 2-parameter model. The tree with the highest log likelihood (−1656.70) is shown. A discrete Gamma distribution was used to model evolutionary rate differences among sites [5 categories (+G, parameter = 0.3354)]. The rate variation model allowed for some sites to be evolutionarily invariable ([+I], 51.51% sites). Bootstrap values >75% in 1000 repetitions are indicated at specific branch nodes. The bar indicates the average number of substitutions per site. The tree was rooted using *Theileria annulata* (KT367871) as the outgroup. Sequences obtained during this study are shown in boldface.

**Figure 5:**
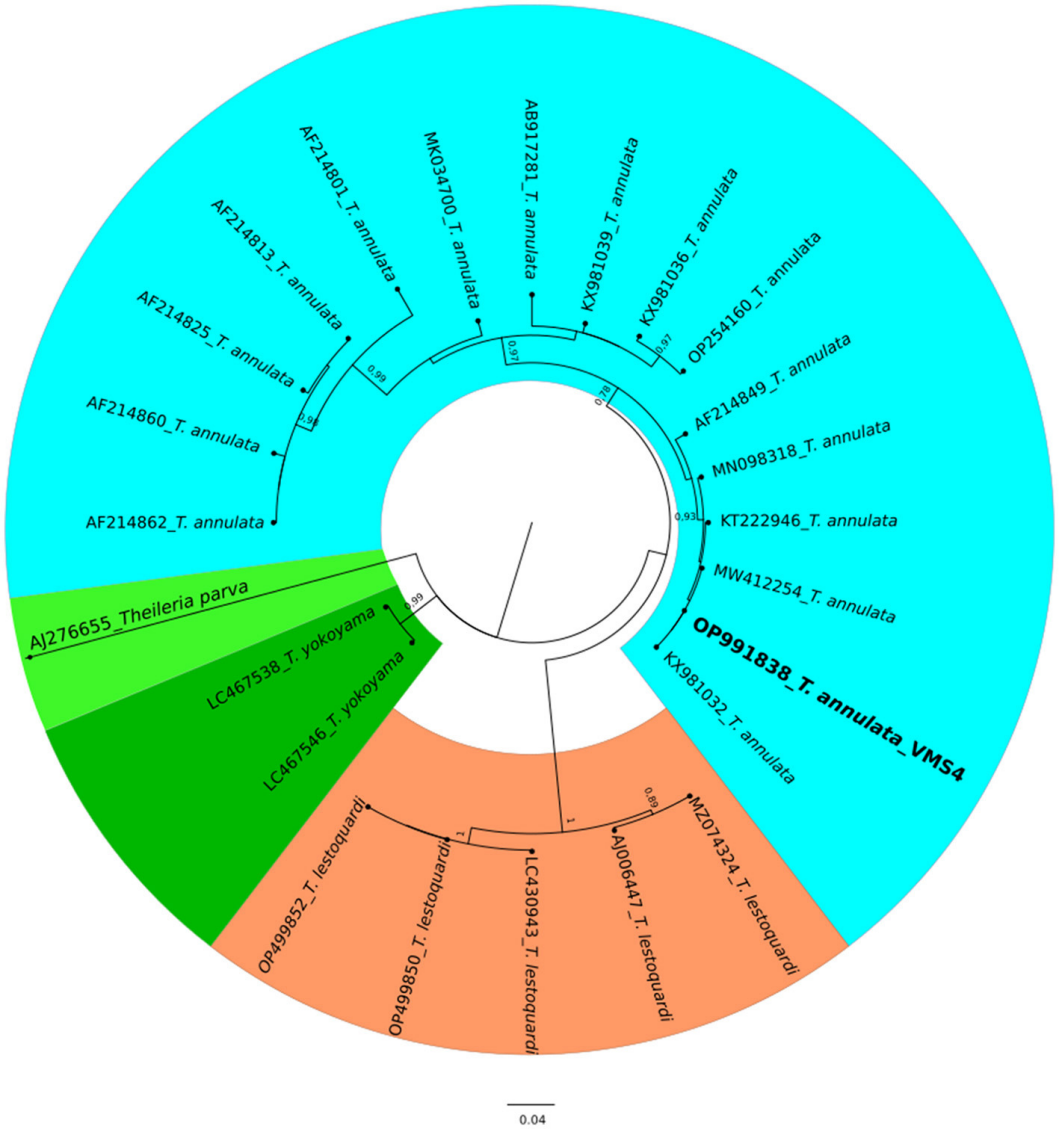
A circular phylogenetic tree of *Theileria annulata* based on the analysis of 24 nucleotide tams1 gene sequences using the Maximum Likelihood method and the Tamura 3-parameter model. The tree depicts the highest log likelihood (−10113.12). Evolutionary rate differences among sites were modeled using a discrete Gamma distribution with 5 categories (+G, parameter = 0.4271). Bootstrap values greater than 75% in 1000 repetitions are indicated at specific branch nodes. The bar represents the average number of substitutions per site. Sequences obtained during this study are highlighted in boldface.

**Figure 6:**
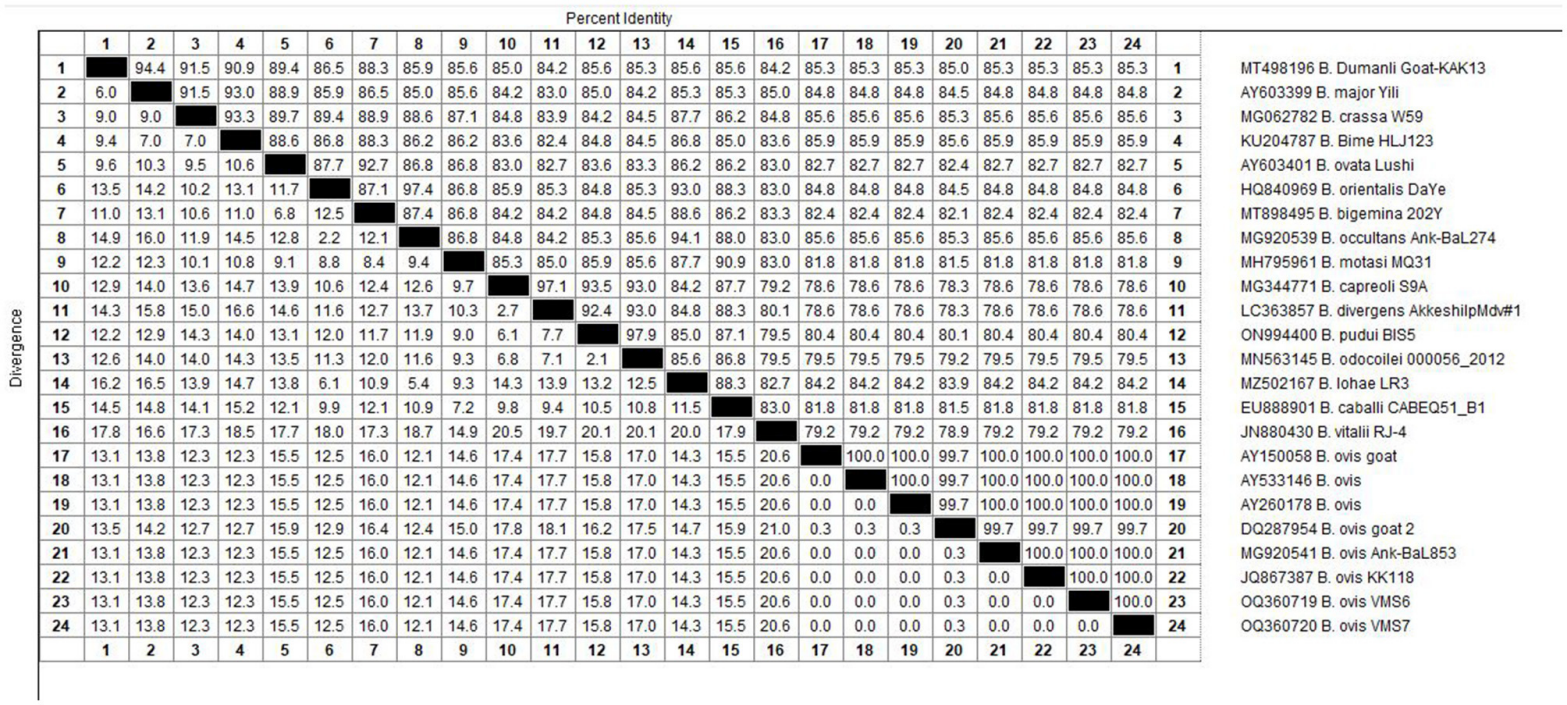
The percentages of identity for the studied isolates of *B. ovis* in sheep related to other isolates worldwide depend on *B. ovis* 18S rRNA gene.

**Figure 7:**
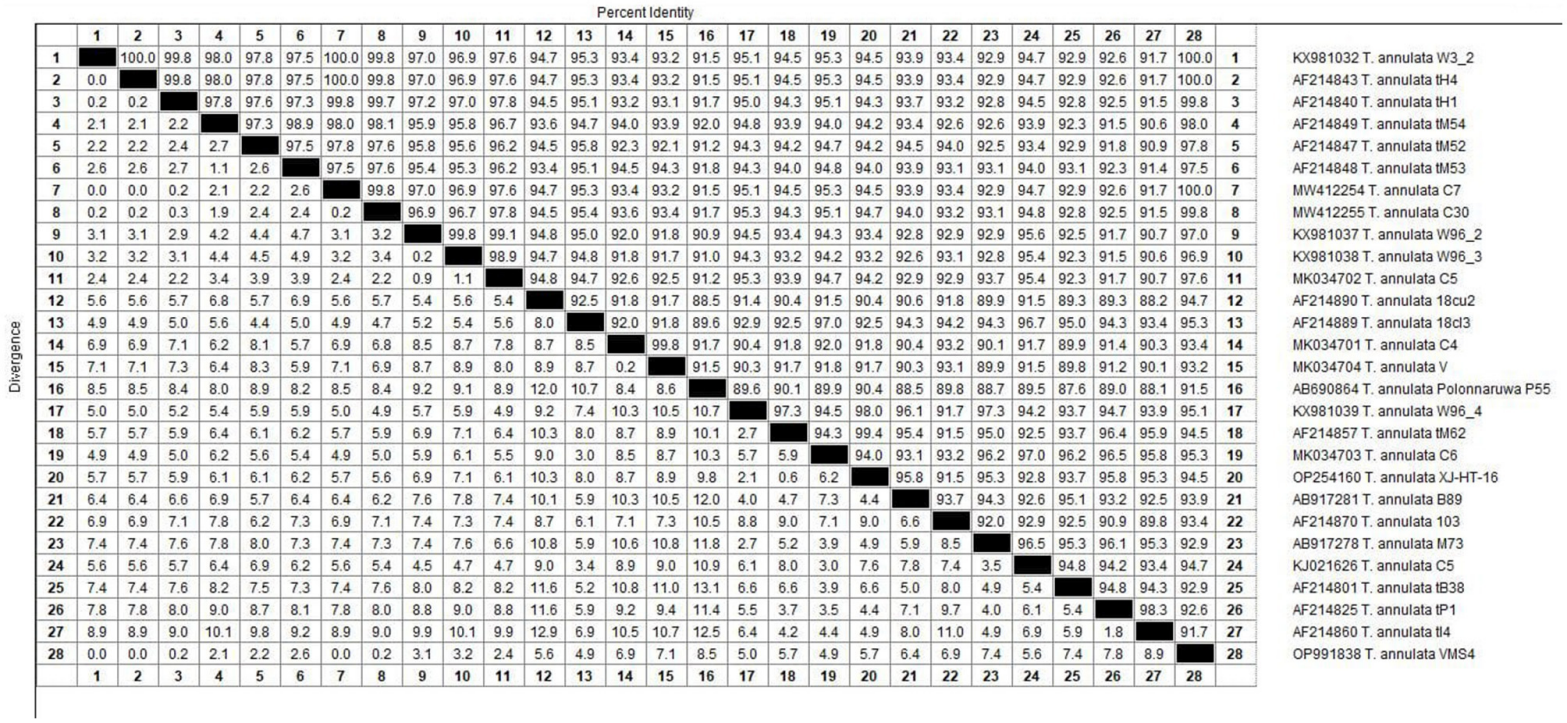
The percentages of identity for the studied isolates of *Theileria annulata* in sheep related to other isolates worldwide depend on *Theileria annulata tams1* gene.

The molecular analysis of the 18S rRNA gene confirmed the presence of *B. ovis* (OQ360720 and OQ360719) with 100% homology to other sequences available in GenBank™. Using the *Tams1* gene for *Theileria*, the presence of *T. annulata* (OP991838) was also confirmed with 100% homology to other sequences accessible in GenBank™. Additionally, the phylogenetic results of this study showed that the presence of *B. ovis* sequences clearly formed a single monophyletic cluster with other sequences of the same species, as depicted in Figures 4. On the other hand, the obtained sequence OP991838 in the present research is clearly differentiated from the rest of the finalized species (*T. parva, T. yokoyama* and *T. lestoquardi*) and integrated into the *T. annulata* cluster (Figure 5). The analysis of intraspecies genetic diversity revealed intriguing findings. For *B. ovis*, the 18S rRNA gene displayed a relatively low level of divergence among sequences (Figure 6). However, the *Tams1* gene for *T. annulata* species exhibited notable intergenetic variability, surpassing the sequences found worldwide, as depicted in Figure 7.

## 4 Discussion

Piroplasmid infection poses a significant threat to sheep populations globally. Considering their veterinary significance and substantial economic implications, understanding the epidemiological pattern of piroplasm's infection is essential for implementing targeted control strategies, including tick control measures, vaccination programs, and improved management practices to reduce the burden of the disease in sheep populations. The current study delves into the exposure and epidemiology of these parasites specifically among sheep populations from Upper Egypt, together with molecular characterization of both parasites. In addition, the study reviewed the prevalence data of *Babesia* spp. and *Theileria* spp. among sheep in Egypt (Table 1). As illustrated, the current study revealed that 176 out of the tested samples tested positive for piroplasmosis using microscopical detection methods, resulting in an overall prevalence of 44%. At national level, a previous study conducted by Hussein et al. (31) in Qena governorate, Upper Egypt, reported an overall prevalence of blood parasites at 38.46% which is slightly lower than our present findings. Conversely, another previous research (33) in the North Coast of Egypt showed a higher prevalence rate of blood parasites (84.80%), surpassing the present reported findings. Regarding the individual prevalence rates reported, the infection rates detected for *Babesia* spp. and *Theileria* spp. were 14% and 30%, respectively. A previous study conducted (4) in Behera governorate (Lower Egypt) reported prevalence rates of 17% for *Babesia* and 20% for *Theileria*, respectively. Another previous study (29) documented presence of *Babesia* spp., with prevalence rates of 23.7%, 23.1%, 21.2%, and 20.6% in the governorates of Beni Suef, Kafr Elsheikh, El-Fayoum, and El-Sharkia, respectively, which surpasses the prevalence rates observed in the current study. Conversely, another study conducted on sheep from Kalubyia governorate (Lower Egypt) revealed a lower incidence of 8.5% for *Babesia* spp. (28). A previous research (56) conducted on sheep from Egypt reported a higher prevalence rate of 50.7% for *Theileria* spp. by Giemsa stained blood film (24–26, 38, 46, 48, 49). Variations in infection prevalence rates may stem from the intricate interactions of factors such as the presence of infected ticks, sanitary and hygienic practices, procedural techniques, immunological status of infected animals, and the absence of veterinary supervision (34, 57, 58). Abiotic factors, particularly temperature and humidity, have a significant impact on the epidemiology of these infections (59). The climatic conditions in the studied area are characterized by mean temperatures throughout the year ranging from 18°C to 40°C, establishing an optimal habitat for the enduring presence of hard ticks. This environment not only facilitates the proliferation of their hosts and the spread of associated blood parasites but also likely contributes to the elevated prevalence rates (60, 61). Concerning the reported clinical signs, the infected sheep exhibited symptoms such as anorexia, anemia, and emaciation. Additionally, hemoglobinuria recorded in sheep infected with *Babesia* spp. Those infected with *Theileria* spp. displayed additional clinical signs such as lacrimation, corneal opacity, and enlarged lymph nodes, consistent with observations reported in the literature (22).

Concerning age, which was explored as a potential risk factor associated with the exposure to those parasites, this study unveiled that the overall prevalence of blood parasites in sheep was non-significantly higher in young animals under 2 years, compared to adult ones over 2 years. In this concern, the study found that *Babesia* spp. infection rates were higher in animals under 2 years compared to those over 2 years, while *Theileria* spp. infection rates were also more prevalent in the younger age group. However, the statistical difference were not significant. In a prior study by Fadly, 2012 (4) in Behera, Upper Egypt, a higher prevalence of both *Babesia* spp. and *Theileria* spp. was higher in sheep above 3 years of age, which contrasts with our findings. Furthermore, Elsayed et al. (33) in North Coast of Egypt concluded that the infection with blood parasites was higher in sheep more than 3 years. Another study (62) in Egypt recorded a higher infection rate in animals aged ≤ 1 year. The discrepancies observed between the current findings and other studies could be attributed to variations in sanitary and hygienic practices, the number of examined animals, variations in immunological resistance, and the impact of local climatic conditions, which may influence the spread of ticks (32).

Regarding sex as a studied individual variable factor, this study found that the overall prevalence of blood parasites in male sheep was significantly higher compared to females, which aligns with earlier studies conducted nationally or internationally (63–66). However, the present findings are in contrast with another reports in Egypt (31, 33, 62). This variation may stem from a notable difference in the sample size of males and females included in our study which could influence the prevalence rates (67). Another contributing factor is management practices in Egypt, where farms typically have few breeding males but a high percentage of young male lambs (68) and the increased susceptibility to infection among females grazing together outdoors (31). It has been demonstrated that the contact of healthy livestock with infected livestock during free grazing is a critical risk factor for increasing the burden and spread of *Theileria* infection (69–71). Furthermore, other previous reports revealed higher occurrence of blood parasites in female as compared to males due to various physiological factors such as pregnancy, parturition, and milk production, may experience increased stress, rendering them more susceptible to infection than males (72). In addition, farm management, microclimatic patterns, tick distribution, host breed, and sampling conditions may account for the variation in prevalence rates of tick-borne pathogens (73, 74). In this study, the prevalence of blood parasites was significantly higher in the cold season 66.1% than in the hot season 15.9%. The infection rate of *Theileria* spp. and *Babesia* spp. was higher in cold season (44.6% and 21.4%, respectively) than in hot season (11.4% and 4.5% respectively). Similarly, another previous study detected higher prevalence of blood parasites in winter when compared to summer (63). The obtained result disagreed with those data recorded (4) in Behera governorate (Lower Egypt), who reported higher infection rate in summer months as compared to winter season. Other studies have documented that there were no differences in the prevalence rate of blood parasites across the analyzed seasons (36). This may be attributed to the presence of ticks and can be explained by the hypothesis that the average temperatures of the studied area during cold months, which are known to provide favorable conditions for the rapid proliferation of ticks and facilitate their easy transmission among susceptible animals (75). These variations can be attributed to geographic and climatic conditions, vector activity, the parasitic status of the investigated area, and the immunological status of the examined animals (4, 76). In this regard, the studied area provides a conducive environment for the development of hard ticks (39). Specifically, the average temperatures in this region during winter might reach 21°C (77, 78), providing optimal conditions for the year-round spread of host vectors and accompanying blood parasites (60, 61).

Regarding the phylogenetic analysis, this study unequivocally identified two bloods parasites species, *B. ovis* through 18S rRNA gene and *T. annulata* by tams1 gene. According to previous studies, a conserved gene of 18S rRNA is a specific target for *Babesia* and *Theileria* species detection by using PCR assay (12, 79, 80), being a sensitive diagnostic tool for ovine piroplasmids. On the other hand, the *Tams1* gene has proven to be a tool to detect new related *Theileria* species (81). Concerning *Theileria*, while blood smear examination remains the primary method for diagnosing, it falls short in species differentiation due to morphological similarities (82). In this work, PCR technique allowed us to identify *T. annulata* in this study. Similarly, a previous work (32) in Egypt detected various *Theilaria* species (*T. ovis, T. annulata* and *T. lestoquardi)*. Experimental infections of sheep and goats with *T. annulata*, the causative agent of tropical theileriosis in cattle, have been documented to induce mild symptoms without piroplasm development. However, previous findings suggest that *T. annulata* can cause mild clinical symptoms in sheep post-experimental infection, and *T. lestoquardi* can similarly affect cattle (83). Precisely, *T. lestoquardi* is considered the primary pathogenic species responsible for malignant ovine theileriosis (84, 85), with many positive samples likely attributed to this species taking into account its mix detection with other species in different studies (9, 76, 86). Nonetheless, another study (87) also identified natural infections of sheep with various species of *Theileria*, known as *T. lestoquardi, T. ovis*, and *T. annulate*, by nested PCR-RFLP. Both infections are transmitted by the same vector, *H. anatolicum*, which is found in Egypt (76) and in many areas, cattle and sheep are raised together, potentially leading to cross-infestation (88, 89). Due to logistical constraints, our study was unable to conduct molecular determinations on all samples. The findings reported here raise the possibility of sheep acting as reservoirs for *T. annulata*, provided both parasites and a competent vector coexist in the same area. Despite this challenge, this study is considered one of the few molecular reports in Egypt confirming *B. ovis*, although confirmation could not be achieved in all samples, highlighting the need for further studies in this area to accurately determine the prevalence of this species in Egypt.

The current study faces several methodological limitations that necessitate careful consideration when interpreting the findings. Initially, screening for both blood parasites involved examining only a single blood film sample per animal using microscopy. While this method remains the gold standard technique for large-scale screening of piroplasmosis, particularly in resource-constrained settings, its accuracy in detection may be compromised, potentially leading to an underestimation of the true infection rate. Secondly, the cross-sectional design of the study is inadequate for monitoring the progression of piroplasmosis and for capturing seasonal variations in infection rates. Thirdly, the findings obtained may not be generalizable to other epidemiological contexts or geographic regions within the same country. Finally, the relatively limited number of samples subjected to molecular characterization may have impacted the precision of estimating the true genetic diversity and frequency of parasite species circulating among the examined sheep.

## 5 Conclusions

The current study has provided significant insights into the prevalence and impact of *Babesia* and *Theileria*, reaffirming their ongoing importance as blood parasites affecting Egyptian sheep in upper Egypt. This investigation emphasizes the critical role of utilizing both traditional microscopy and cutting-edge molecular methods for the precise detection and characterization of piroplasmosis. Given the above-mentioned findings, of particular note is the apparent rise in the circulation of piroplasmosis, a trend likely worsened by the expanding populations of the tick vectors. Furthermore, the study sheds light on the current epidemiological landscape of piroplasmosis in the Egyptian environment. By addressing the spread of these parasites, informed actions can be taken to mitigate the associated diseases, safeguarding the health and wellbeing of both livestock and the population at large. These findings serve as a call to action, emphasizing the importance of ongoing surveillance and strategic interventions to manage and minimize the impact of *Babesia* and *Theileria* infections in Egypt. This underscores the urgent necessity for additional investigations centered on these vectors, signaling a critical domain for future research endeavors and intervention initiatives. Further studies with larger sample sizes, diverse small ruminants, and additional epidemiological data are also necessary to comprehensively evaluate the associated risk factors for infection. Such knowledge would be helpful for governmental authorities to develop and implement effective control measures.

## Data availability statement

The original contributions presented in the study are publicly available. This data can be found at: https://www.ncbi.nlm.nih.gov/nuccore/; OP984151.1.

## Ethics statement

The animal studies were approved by Research Ethics Committee of the Faculty of Veterinary Medicine, Assiut University, under the designation 06/2023/0130. The studies were conducted in accordance with the local legislation and institutional requirements. Written informed consent was obtained from the owners for the participation of their animals in this study.

## Author contributions

AD: Investigation, Methodology, Project administration, Supervision, Validation, Visualization, Writing—original draft, Writing—review & editing. FA-A: Conceptualization, Data curation, Formal analysis, Investigation, Methodology, Software, Validation, Writing—original draft, Writing—review & editing. SM: Conceptualization, Methodology, Software, Supervision, Validation, Visualization, Writing—original draft, Writing—review & editing. AG: Conceptualization, Data curation, Formal analysis, Investigation, Software, Supervision, Validation, Visualization, Writing—original draft, Writing—review & editing. FO: Conceptualization, Data curation, Investigation, Methodology, Software, Supervision, Validation, Visualization, Writing—original draft, Writing—review & editing. FE: Data curation, Formal analysis, Investigation, Software, Validation, Writing—review & editing. EH: Data curation, Formal analysis, Software, Validation, Visualization, Writing—review & editing. NA: Conceptualization, Data curation, Formal analysis, Software, Validation, Writing—review & editing. HA: Data curation, Formal analysis, Funding acquisition, Investigation, Software, Validation, Writing—review & editing. AA: Data curation, Formal analysis, Software, Validation, Writing—review & editing. DB-B: Data curation, Formal analysis, Software, Writing—review & editing. EE: Conceptualization, Data curation, Formal analysis, Funding acquisition, Investigation, Methodology, Resources, Validation, Writing—original draft, Writing—review & editing.
