# Supplementary material for: Microscopic and molecular detection of piroplasms among sheep in Upper Egypt
Source: Front Vet Sci. 2024 May 27;11:1373842. doi: 10.3389/fvets.2024.1373842 (PMC11163088; doi:10.3389/fvets.2024.1373842)
Supplement: Supplementary file 1 [file Table_1.DOCX]

**Supplementary Table 1**. Oligonucleotides were utilized for the molecular identification of blood parasites in this study.

| **Target gene** | **Primer sequence(5'-3')** | **Size (bp)** | **Reference** |
| --- | --- | --- | --- |
| *Babesia 18S rRNA* | GTCTTGTAATTGGAATGATGGTGAC | 340 | (47) |
|  | ATGCCCCCAACCGTTCCTATTA |  |  |
| *Theileria annulata tams1* | GTAACCTTTAAAAACGT | 721 | (46) |
|  | GTTACGAACATGGGTTT |  |  |
