# Supplementary material for: Microscopic and molecular detection of piroplasms among sheep in Upper Egypt
Source: Front Vet Sci. 2024 May 27;11:1373842. doi: 10.3389/fvets.2024.1373842 (PMC11163088; doi:10.3389/fvets.2024.1373842)
Supplement: Supplementary file 2 [file Table_2.DOCX]

**Supplementary Table 2.** PCR cycling conditions used for the molecular identification of *Babesia* and *Theileria* species investigated in this study.

|  |  | **Temperature and time** | | | | |  |  |  |
| --- | --- | --- | --- | --- | --- | --- | --- | --- | --- |
| **Target organism** | **Locus** | | **Initial denaturation** | **Denaturation** | **Annealing** | **Extension** | **No. cycles** | **Final extension** | **Reference** |
| *Babesia* and *Theileria* spp. | *Babesia 18S rRNA, and*  *Theileria annulata tams1* | | 94°C 5 min | 94°C 30 s | 55°C 40 s | 72°C 40 s | 35 | 72°C 10 min | (46, 47) |
|  |  | |  |  |  |  |  |  |  |
